# Supplementary material for: CAFT: A Compositional Log-Linear Model for Microbiome Data with Zero Cells
Source: bioRxiv. 2025 Dec 1:2025.11.26.690468. Preprint. [Version 1] doi: 10.1101/2025.11.26.690468 (PMC12694591; doi:10.1101/2025.11.26.690468)
Supplement: 1 [file NIHPP2025.11.26.690468V1-supplement-1.pdf]

bioRxiv preprint doi: https://doi.org/10.1101/2025.11.26.690468; this version posted December 1, 2025. The copyright holder for this preprint (which was not certified by peer review) is the author/funder, who has granted bioRxiv a license to display the preprint in perpetuity. It is made available under aCC-BY 4.0 International license.

S1: The Cox variance estimator

The Cox variance estimator based on derives from a Martingale representation of the Cox model. Dropping the superscript for taxon (j), the Martingale representation of S is

$$S = \sum_{i=1}^{N} \int_{-\infty}^{e_i} \Gamma_0(e) \left(X_{i\cdot} - \frac{\Gamma_1(e)}{\Gamma_0(e)} \right) dM_i(e) \tag{13}$$

where

$$\Gamma_k(e) = \sum_{i=1}^{N} I(e_i \geq e) X_{i\cdot}^{\otimes k} \tag{14}$$

and $dM_i(e) = dN_i(e) - Y(e)d\Lambda(e)$ and $N_i(e) = I(e_i \leq e)$, where Λ is the cumulative hazard and Y is the at-risk indicator. Then, S is a weighted version of the Cox score with weight $\Gamma_0(e)$. We then define $\widehat{S}_i$ as

$$\widehat{S}_i = \Delta_i[\Gamma_0(e_i)X_{i\cdot} - \Gamma_1(e_i)] - \sum_{\{i' | e_{i'} \leq e_i\}} [\Gamma_0(e_{i'})X_{i'\cdot} - \Gamma_1(e_{i'})]\delta\widehat{\Lambda}(e_{i'}) \tag{15}$$

where $\delta\widehat{\Lambda}(e)$ is the jump in the estimated cumulative hazard Λ at residual e. Then, the Cox variance proposed by Zeng and Lin (19) is

$$\widehat{V}_j(\beta_j) = \frac{1}{N} \sum_i \widehat{S}_i \widehat{S}_i^T. \tag{16}$$

DRAFT

bioRxiv preprint doi: https://doi.org/10.1101/2025.11.26.690468; this version posted December 1, 2025. The copyright holder for this preprint (which was not certified by peer review) is the author/funder, who has granted bioRxiv a license to display the preprint in perpetuity. It is made available under aCC-BY 4.0 International license.

S2: Additional Simulations

To assess the performance of our variance estimators for the AFT model, we simulated survival time using the following equation,

$$t_i = \gamma + \beta_1 x_{i1} + \beta_2 x_{i2} + \epsilon_i, \quad i = 1, \ldots, n.$$

Here, x_{i1} was the variable of interest and x_{i2} served as the adjustment variable. We generated x_{i1} from a standard normal distribution and induced correlations between covariates via $x_{i2} = \rho x_{i1} + \sqrt{1-\rho^2} z_{i2}$ with $z_{i2} \sim N(0,1)$. The correlation parameter $\rho \in \{-0.5, 0, 0.3, 0.5\}$ controls the direction and strength of the confounding effect. Error terms ϵ_i were generated independently from four distributions: $N(0,1)$, Exponential(1), Cauchy(0,1) and Weibull($k = 1.5$, $\lambda = 1$), each centered to have mean zero. The censoring time c_i was drawn as $c_i \sim N(\mu_c, 1)$. The observed survival time was $\tau_i = \min(t_i, c_i)$ with censoring indicator $\Delta_i = I(t_i < c_i)$. We considered two censoring scenarios by choosing $\mu_c = -0.9$ to yield moderate censoring (around 60-70%, Table S1) and choosing $\mu_c = -3.3$ to yield high censoring (around 80-98%, Table S2). The detailed average censoring proportions for different error distributions appear in the tables. For type-I error evaluation, we fixed $\beta_1 = 0$, $\beta_2 = 1$, and $\gamma = 0$. Each design was replicated 10,000 times with $n \in \{100, 200, 500\}$. In each replicate, we tested $H_0 : \beta_1 = 0$ using the proposed CAFT variance estimator for the AFT model and, for comparison, a COX variance estimator, and recorded the empirical rejection proportion at $\alpha = 0.05$.

Distribution	Censoring Proportion	n	Method	ρ			
				-0.5	0	0.3	0.5
Normal	$\sim 70\%$	100	CAFT	0.0482	0.0458	0.0481	0.0498
			COX	0.0566	0.0543	0.0529	0.0569
		200	CAFT	0.0490	0.0501	0.0486	0.0524
			COX	0.0530	0.0518	0.0536	0.0573
		500	CAFT	0.0508	0.0528	0.0498	0.0517
			COX	0.0539	0.0525	0.0506	0.0524
Exponential	$\sim 70\%$	100	CAFT	0.0496	0.0441	0.0509	0.0493
			COX	0.0584	0.0566	0.0571	0.0586
		200	CAFT	0.0476	0.0487	0.0487	0.0506
			COX	0.0543	0.0538	0.0521	0.0533
		500	CAFT	0.0483	0.0485	0.0481	0.0456
			COX	0.0506	0.0500	0.0486	0.0471
Cauchy	$\sim 60\%$	100	CAFT	0.0535	0.0496	0.0478	0.0504
			COX	0.0650	0.0646	0.0647	0.0673
		200	CAFT	0.0500	0.0491	0.0508	0.0509
			COX	0.0586	0.0577	0.0589	0.0589
		500	CAFT	0.0503	0.0529	0.0533	0.0515
			COX	0.0526	0.0549	0.0557	0.0531
Weibull	$\sim 72\%$	100	CAFT	0.0451	0.0492	0.0459	0.0477
			COX	0.0568	0.0598	0.0582	0.0561
		200	CAFT	0.0510	0.0524	0.0558	0.0524
			COX	0.0567	0.0584	0.0603	0.0571
		500	CAFT	0.0470	0.0511	0.0517	0.0484
			COX	0.0477	0.0540	0.0541	0.0513

DRAFT

Table S1. Empirical type-I error ($\alpha = 0.05$) for testing $H_0 : \beta_1 = 0$ using the proposed CAFT variance–covariance estimator for the AFT model and a Cox-based variance estimator (COX). Results are shown by error distribution (Normal, Exponential, Cauchy, Weibull), sample size ($n = 100, 200, 500$), confounding correlation $\rho \in \{-0.5, 0, 0.3, 0.5\}$, and the moderate average censoring proportion (around 60 - 70 %). Each entry is the rejection proportion over 10,000 simulations.

Across all designs, the proposed CAFT variance estimator maintained nominal type-I error, while the COX estimator was increasingly liberal as censoring intensified. Under moderate censoring (around 60 - 70 % in Table S1), CAFT stayed close to 0.05 for every error distribution and $n = 100, 200, 500$, including the heavy-tailed Cauchy case. COX was generally near nominal but tended to over-reject, most noticeably with Cauchy errors. Under high censoring (around 80 - 98 % in Table S2), CAFT again hovered around 0.05 across scenarios, roughly from 0.047 to 0.052, whereas COX showed marked inflation for Exponential and Weibull errors, about 0.12 to 0.16 at $n = 100$ and 0.08 to 0.13 at $n = 200$. For $n = 500$, the type I error inflation reduced (about 0.05 to 0.06) but is still above CAFT. For Normal errors, COX remained near nominal under high censoring,

bioRxiv preprint doi: https://doi.org/10.1101/2025.11.26.690468; this version posted December 1, 2025. The copyright holder for this preprint (which was not certified by peer review) is the author/funder, who has granted bioRxiv a license to display the preprint in perpetuity. It is made available under aCC-BY 4.0 International license.

and for Cauchy, it was slightly inflated when the sample size is small ($n = 100$ and 200). Variations across the correlation parameter ρ were minor relative to the effects of censoring level, error distribution, and sample size.

Distribution	Censoring Proportion	n	Method	ρ = -0.5	ρ = 0	ρ = 0.3	ρ = 0.5
Normal	$\sim 97\%$	100	CAFT	0.0493	0.0501	0.0475	0.0466
			COX	0.0552	0.0519	0.0537	0.0572
		200	CAFT	0.0490	0.0503	0.0492	0.0501
			COX	0.0286	0.0320	0.0288	0.0312
		500	CAFT	0.0524	0.0502	0.0488	0.0497
			COX	0.0495	0.0523	0.0515	0.0501
Exponential	$\sim 98\%$	100	CAFT	0.0479	0.0404	0.0429	0.0467
			COX	0.1516	0.1390	0.1401	0.1504
		200	CAFT	0.0520	0.0506	0.0474	0.0515
			COX	0.0883	0.0808	0.0887	0.0890
		500	CAFT	0.0508	0.0506	0.0527	0.0482
			COX	0.0556	0.0479	0.0593	0.0525
Cauchy	$\sim 81\%$	100	CAFT	0.0457	0.0471	0.0474	0.0474
			COX	0.0674	0.0661	0.0666	0.0720
		200	CAFT	0.0483	0.0493	0.0479	0.0490
			COX	0.0617	0.0609	0.0600	0.0589
		500	CAFT	0.0485	0.0487	0.0483	0.0490
			COX	0.0557	0.0541	0.0543	0.0537
Weibull	$\sim 98\%$	100	CAFT	0.0401	0.0357	0.0377	0.0437
			COX	0.1562	0.1424	0.1430	0.1532
		200	CAFT	0.0494	0.0450	0.0413	0.0473
			COX	0.1224	0.1211	0.1184	0.1286
		500	CAFT	0.0511	0.0475	0.0485	0.0512
			COX	0.0554	0.0494	0.0521	0.0535

Table S2. Empirical type-I error ($\alpha = 0.05$) for testing $H_0 : \beta_1 = 0$ using the proposed CAFT variance–covariance estimator for the AFT model and a Cox-based variance estimator (COX). Results are shown by error distribution (Normal, Exponential, Cauchy, Weibull), sample size ($n = 100, 200, 500$), confounding correlation $\rho \in \{-0.5, 0, 0.3, 0.5\}$, and the high average censoring proportion (around 80 - 98 %). Each entry is the rejection proportion over 10,000 simulations.

DRAFT

bioRxiv preprint doi: https://doi.org/10.1101/2025.11.26.690468; this version posted December 1, 2025. The copyright holder for this preprint (which was not certified by peer review) is the author/funder, who has granted bioRxiv a license to display the preprint in perpetuity. It is made available under aCC-BY 4.0 International license.

S3: Supplementary Figures

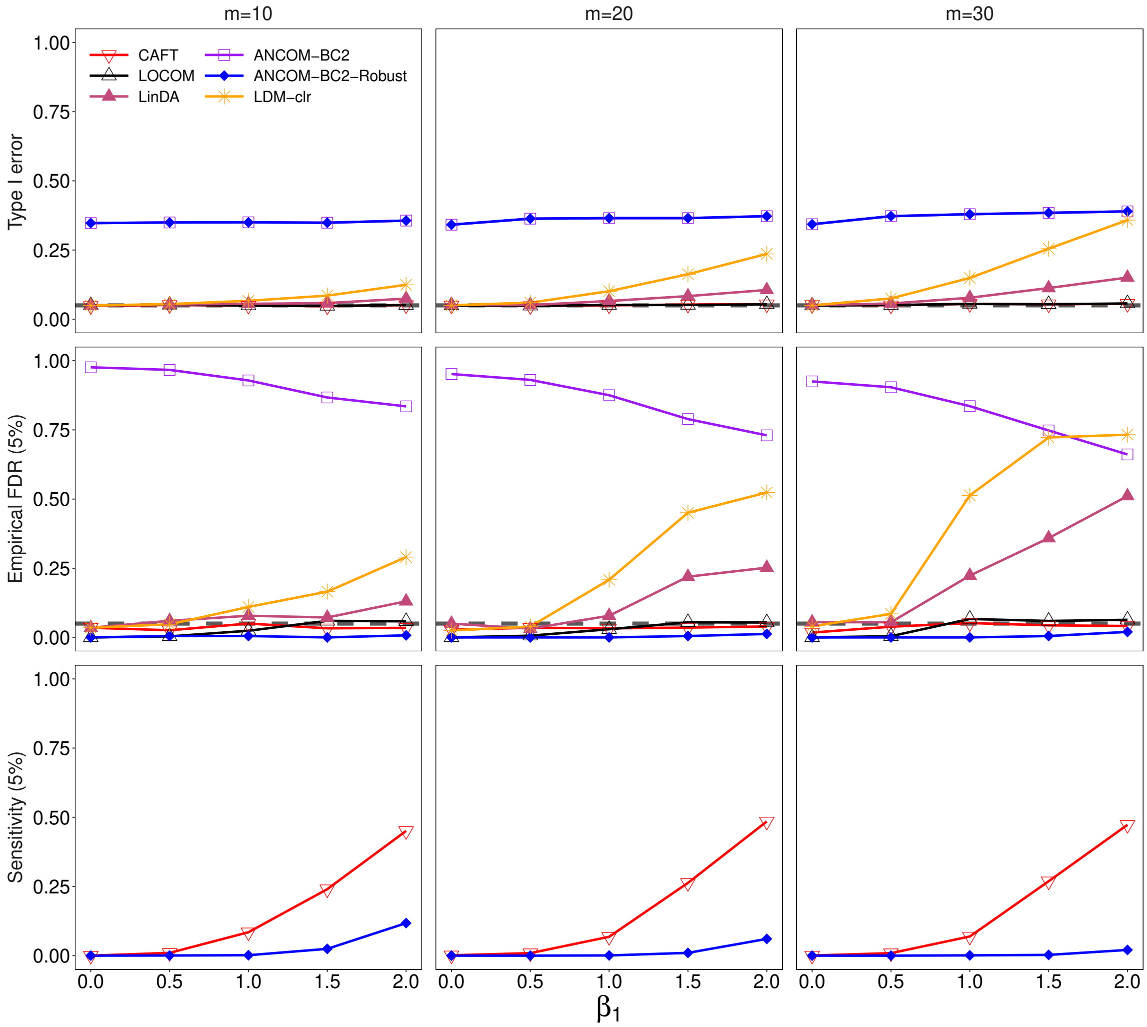

Fig. S1. Results from the MIDASim simulation: x_1 and x_2 are both binary, bias $(b = 1)$, taxa filtered at 6%, n = 100. The gray dashed line indicates the nominal level Type I error of 0.05 in the first row. Numbers in parentheses of row names represent the FDR cutoffs applied during the evaluation.

bioRxiv preprint doi: https://doi.org/10.1101/2025.11.26.690468; this version posted December 1, 2025. The copyright holder for this preprint (which was not certified by peer review) is the author/funder, who has granted bioRxiv a license to display the preprint in perpetuity. It is made available under aCC-BY 4.0 International license.

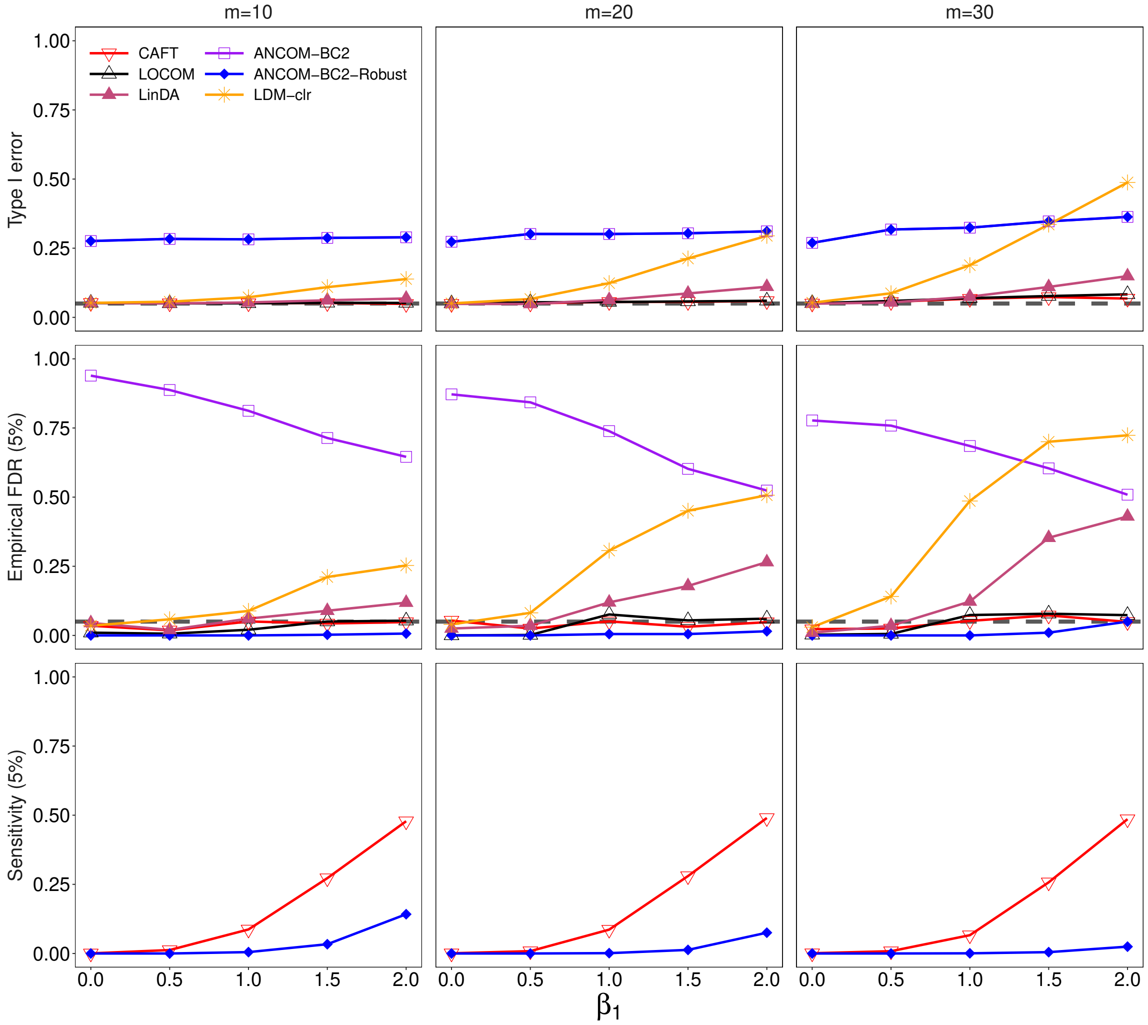

Fig. S2. Results from the MIDASim simulation: x_1 and x_2 are both binary, bias ($b = 2$), taxa filtered at 6%, n = 100. The gray dashed line indicates the nominal level Type I error of 0.05 in the first row. Numbers in parentheses of row names represent the FDR cutoffs applied during the evaluation.
